# Long non‐coding RNA HEIH suppresses the expression of *TP53* through enhancer of zeste homolog 2 in oesophageal squamous cell carcinoma

**DOI:** 10.1111/jcmm.15673

**Published:** 2020-07-30

**Authors:** XinYu Ding, Chen Qi, Jie Min, ZhiFei Xu, KeNan Huang, Hua Tang

**Affiliations:** ^1^ Department of Minimally Invasive Thoracic Surgery Center Shanghai Changzheng Hospital Second Military Medical University Shanghai China; ^2^ Department of Cardiothoracic Surgery Jinling Hospital Medical School of Nanjing University Nanjing China

**Keywords:** lncRNA‐HEIH, long non‐coding RNA, oesophageal squamous cell carcinoma, TP53, zeste homolog 2

## Abstract

It is increasingly evident that the molecular and biological functions of long non‐coding RNAs (lncRNA) are vital for understanding the molecular biology and progression of cancer. The lncRNA‐HEIH, a newly identified lncRNA, has been demonstrated to be up‐regulated in hepatocellular cancer. However, little is known about its role in oesophageal squamous cell carcinoma (ESCC). In the present study, an obvious up‐regulation of lncRNA‐HEIH was observed in ESCC compared to the adjacent normal tissues. Meanwhile, patients with high expression of lncRNA‐HEIH have significantly poorer prognosis than those with low expression. We further found that lncRNA‐HEIH was associated with enhancer of zeste homolog 2 (EZH2) and that this association led to the repression of *TP53*. These findings indicate that lncRNA‐HEIH may serve as a prognostic marker and a potential therapeutic target for ESCC.

## 1 INTRODUCTION

Oesophageal cancer is one of the least studied and deadliest cancers worldwide because of its extremely aggressive nature and poor survival rate.^1^, ^2^ Oesophageal squamous cell carcinoma (ESCC), the major histologic form of oesophageal cancer, dominates in most parts of the world, especially in northern China with a particularly high incidence rate.^3^, ^4^ Like other solid tumours, inactivation of tumour suppressor genes (TSG) and activation of oncogenes occur during the progression of ESCC.^5^, ^6^ Unfortunately, despite efforts to improve its prognosis, the overall survival rate of patients with ESCC is still unsatisfactory, mainly because of the advanced stage at initial diagnosis and the deficiency of efficacious therapies.^7^ Therefore, it is essential to identify new sensitive and specific molecular markers for early diagnosis and therapeutic targets.

Long non‐coding RNAs (lncRNAs) are non‐protein‐coding RNAs that pervasively exist throughout eukaryotic genomes.^8^ The lncRNAs play important roles in dosage compensation, imprinting and homeotic gene expression.^9^, ^10^ Increasing evidence suggests that lncRNAs regulate key pathways in cancer invasion and metastasis.^11^, ^12^ One example of such oncogenic lncRNAs is HEIH transcript which is overexpressed in hepatitis B virus‐related hepatocellular carcinoma.^13^ Furthermore, the extent of HEIH gene expression in primary hepatocellular carcinoma is a powerful indicator of treatment outcomes. The functions of lncRNA‐HEIH in the progression of other cancers have not been fully elucidated.

In our study, we investigated whether expression of the HEIH gene was associated with ESCC growth and metastasis. Accordingly, the level of HEIH in ESCC tissues was detected and its potential relationship with clinical pathologic parameters and tumour recurrence was analysed. The role of lncRNA‐HEIH in growth and metastasis during progression of ESCC was also studied both in vitro and in vivo.

## 2 MATERIALS AND METHODS

### 2.1 Patients and cell lines

Frozen tumour samples from ESCC patients were randomly obtained from Shanghai Changzheng Hospital (Shanghai, PR China) with informed consent. Studies using human tissue were reviewed and approved by the Committees for Ethical Review of Research involving Human Subjects of Second Military Medical University (Shanghai, China). Total RNA was extracted with TRIzol reagent (Invitrogen, Carlsbad, CA) according to the manufacturer's instructions. The Eca109, TE13 and HEEpiC cell lines were cultured at 37°C in an atmosphere containing 5% CO2 in Dulbecco's modified Eagle's medium supplemented with 10% foetal bovine serum. The collection of human sample was approved by the Ethics Committee of Changzheng Hospital.

### 2.2 Real‐time PCR (RT‐PCR) and Western blot analysis

We employed RT‐PCR analyzation to measure the expression levels of RNAs. Total RNA was isolated using TRIzol (Invitrogen), and genomic DNA was removed by RNase‐free DNase. First‐strand cDNA was generated with the Prime Script RT reagent kit (Takara, Dalian, PR China). Step One™ Real‐Time PCR System (Applied Biosystems, Foster City, USA) and gene‐specific primers were applied for RT‐PCR. Gene expression in each sample was normalized to 18S rRNA expression. Significance was determined by taking the average of the 18S rRNA‐normalized 2^–ΔΔCT^ values. The gene‐specific primers can be seen details in Table S1.

In Western blotting analysis, antibody dilutions were 1:1000 for PCNA (Cell signaling Technology, Boston, USA) and 1:5000 for β‐actin (Sigma‐Aldrich).

### 2.3 Lentivirus infection and construction of cell lines stably expressing LncRNA‐HEIH

ESCC cells were infected with concentrated virus at a multiplicity of infection of 100, 75 and 50 in the presence of 10 μg/ml polybrene (Sigma‐Aldrich, St. Louis, MO). The supernatant was removed after 24 hours and replaced with complete culture medium. Infected cells were confirmed by RT‐PCR 96 hours after infection. The lncRNA‐HEIH siRNAs are presented in Table S1.

### 2.4 Cell proliferation assay

A total of approximately 3.0 × 10^3^ ESCC cells were plated in 96‐well plates. Cell proliferation was assessed using the Cell Counting Kit‐8 (Beyotime, Jiangsu, China) according to the manufacturer's protocol. All of the experiments were performed in triplicate. The cell proliferation curves were plotted based on the absorbance at each time point.

### 2.5 Small interfering RNA (siRNA)

To inhibit EZH2, 50 nmol/L EZH2 siRNA (Santa cruze) was transfected into ESCC cells using Lipofectamine 2000 reagent according to the manufacturer's instructions. Cells transfected by the transfection agent with no siRNA (Mock) and with scramble‐controlled siRNA (Negative control) were employed as controls. The cells were harvested 48 hours after transfection.

### 2.6 Nude mouse ESCC model

All of the male Balb/c nude mice used in this experiment were purchased from Shanghai Experimental Animal Center of Chinese Academy of Sciences (Shanghai, PR China) and received humane care. To figure out the effects of lncRNA‐HEIH on cancer cell dynamics in vivo, ESCC cells were transfected with pCMV‐luciferase (Promega) and selected with neomycin (800 µg/ml). ESCC cells stably expressing luciferase were infected with lncRNA‐HEIH overexpression virus as described above. Five million lncRNA‐HEIH‐up‐regulated cells (firefly luciferase‐labelled ESCC cells) and control cells were injected into the double flank of nude mice. For firefly luciferase‐labelled ESCC cells, tumour growth was monitored using the IVIS@ Lumina II system (Caliper Life Sciences, USA) 10 min after intraperitoneal injection of 4.0 mg luciferin (Gold Biotech) in 50 μl saline. The experimental protocols were approved by the Ethical Committee of the Second Military Medical University.

### 2.7 RNA Immunoprecipitation

We performed RNA immunoprecipitation (RIP) experiments with the Magna RIP™ RNA‐Binding Protein Immunoprecipitation Kit (Millipore, USA)according to the manufacturer's instructions. The EZH2 antibodies used for RIP were clone AC22 (17‐662, Millipore). The co‐precipitated RNAs were detected by reverse transcription PCR. Total RNAs (input controls)and isotype controls were assayed simultaneously to demonstrate that the detected signals were from RNAs specifically binding to EZH2 (n = 3 for each experiment). The gene‐specific primers used for detecting lncRNA‐HEIH could refer to Table S2.

### 2.8 Chromatin immunoprecipitation

Chromatin immunoprecipitation (ChIP) was performed with the EZ ChIP™ Chromatin Immunoprecipitation Kit (Millipore Bedford, MA, USA) in line with its manual. Briefly, cross‐linked chromatin was sonicated into fragments with 200‐1000 bp. The chromatin was immunoprecipitated by anti‐Ezh2 (clone AC22) and anti‐H3K27me3 (Millipore). Normal mouse IgG was applied as a negative control. Quantitative PCR was conducted using SYBR Green Mix (Takara Bio, Otsu, Japan). Primer sequences are listed in Table S2.

### 2.9 Microarray analysis

Gene expression profiles of the Eca109 cells with or without lncRNA‐HEIH overexpression were determined by Phalanx human One Array microarrays (HOA 6.1) following the manufacturer's instructions.

Gene‐gene interaction network was constructed based on the data of differentially expressed genes. Network maps were constructed via Java which allows users to build and analyse molecular networks. For instance, if there is confirmative evidence that two genes interact with each other, an interaction edge is assigned between them. The considered evidence is from the interaction database from KEGG. Networks are stored and presented as graphs, where nodes are mainly genes (protein, compound, etc) and edges represent relation types between the nodes, for example activation or phosphorylation. The graph nature of networks raised our interest to view the networks as a powerful tool implemented in R.

To investigate the global network, we computationally identify the critical nodes. To this end, we defined the connectivity (also known as degree) as the sum of connection strengths with the other network genes:

$$K_{i}=\sum_{u\neq i}a_{\mathrm{ui}}$$

In gene networks, the connectivity measures how correlated a gene is with all other network genes. For a gene in the network, the number of source genes of a gene is called the indegree of the gene and the number of target genes of a gene is its outdegree. The character of genes is described by betweenness centrality measures reflecting the importance of a node in a graph relative to other nodes. For a graph G:(V, E) with n vertices, the relative betweenness centrality *C_B_*′ (*v*) is defined.

### 2.10 Statistical evaluation

All the statistical analyses were conducted by SPSS version 17.0 software. For comparisons, one‐way analyses of variance, Fisher's exact tests, chi‐squared tests and two‐tailed Student's *t* tests were performed as appropriate. The Kaplan‐Meier method was included to evaluate the cumulative survival probability was evaluated using the Kaplan‐Meier method, and differences were assessed using the log‐rank test.

## 3 RESULTS

### 3.1 LncRNA‐HEIH expression is up‐regulated in human ESCC tissues

**Figure 1:**
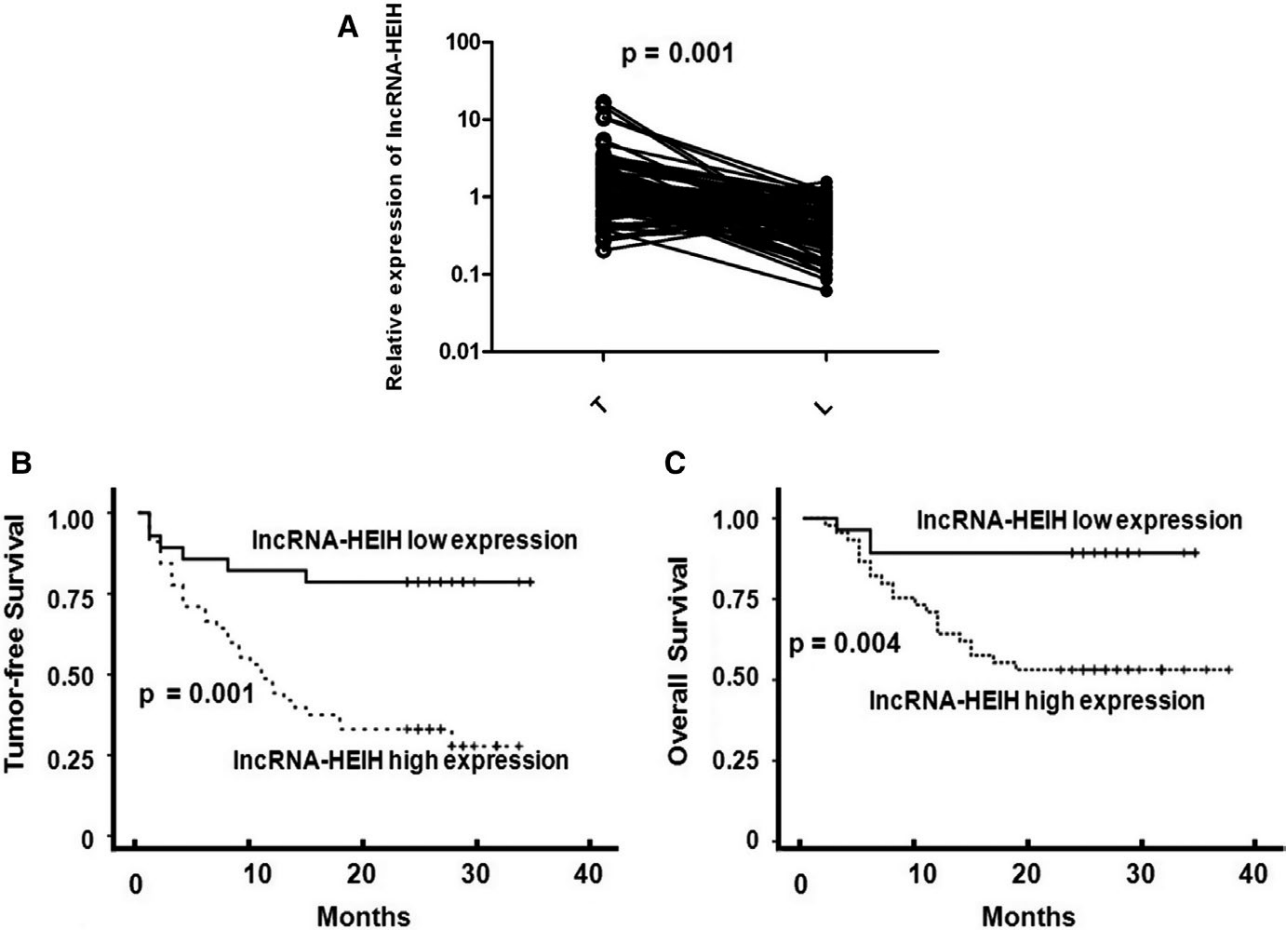
Up‐regulation of lncRNA‐HEIH Correlates with Poor Survival in ESCC Patients. (A) The lncRNA‐HEIH transcript expression level was determined by SYBR‐green real‐time PCR in ESCC (T) and paired non‐tumour tissues (L) (n = 91). Shown are Kaplan‐Meier survival curves of tumour‐free survival (B) and overall survival (C), according to the expression levels of lncRNA‐HEIH in each tumour sample; the median value in each cohort was chosen as the cut‐off point. β‐actin was used as the internal control of transcript expression

Quick RT‐PCR analysis was used to measure lncRNA‐HEIH level in 91 ESCC tissues and normal counterparts. The expression of lncRNA‐HEIH was significantly up‐regulated in ESCC tissues (Figure 1A). Furthermore, correlation analysis of lncRNA‐HEIH expression with clinical pathological features of ESCC patients revealed a significant association between lncRNA‐HEIH up‐regulation and advanced pathological stage, tumour invasion and lymph node metastasis (Table 1). However, lncRNA‐HEIH expression was not correlated with histological subtype and gender. Kaplan‐Meier survival analysis and log‐rank tests on patients postoperative survival were performed to further evaluate the correlation between lncRNA‐HEIH expression and ESCC patient prognosis. According to the median ratio of relative lncRNA‐HEIH expression in tumour tissues, the 91 ESCC patients were classified into two groups: High‐lncRNA‐HEIH group (n = 46, lncRNA‐HEIH expression ratio ≥ mean ratio) and Low‐lncRNA‐HEIH group (n = 45, lncRNA‐HEIH expression ratio < mean ratio). The Kaplan‐Meier survival curve showed that patients with high‐lncRNA‐HEIH expression levels evidently had shorter tumour‐free and overall survival time than those with low levels (Figure 1B and C). These findings support the hypothesis that high level of lncRNA‐HEIH expression plays a key role in ESCC progression.

**Table 1:** Association of lncRNA‐HEIH up‐regulation with clinicopathologic features in ESCCs

Clinical features	Number	lncRNA‐HEIH up‐regulation	*P*
Age, years			
≤60	53	40 (74.5%)	.926
>60	38	29 (76.3%)	
Gender			
Male	49	35 (71.4%)	.290
Female	42	34 (81.0%)	
Differentiation			
Well	18	12 (66.7%)	.614
Moderate	46	36 (78.3%)	
Poor	27	21 (77.8%)	
Tumour invasion			
T_1_	5	1 (20.0%)	.017^a^
T_2_	12	5 (41.7%)	
T_3_	37	24 (64.9%)	
T_4_	37	29 (78.4%)	
Lymph node metastasis			
N_0_	56	36 (64.3%)	.001^a^
N_1_	35	33 (94.3%)	
Clinical stage			
Early (I‐II)	60	39 (65%)	.001^a^
Advanced (III‐IV)	31	30 (96.8%)	

^a^ Statistical significance (*P* < .05) is shown.

### 3.2 LncRNA‐HEIH promotes the cell proliferation in ESCC cells

**Figure 2:**
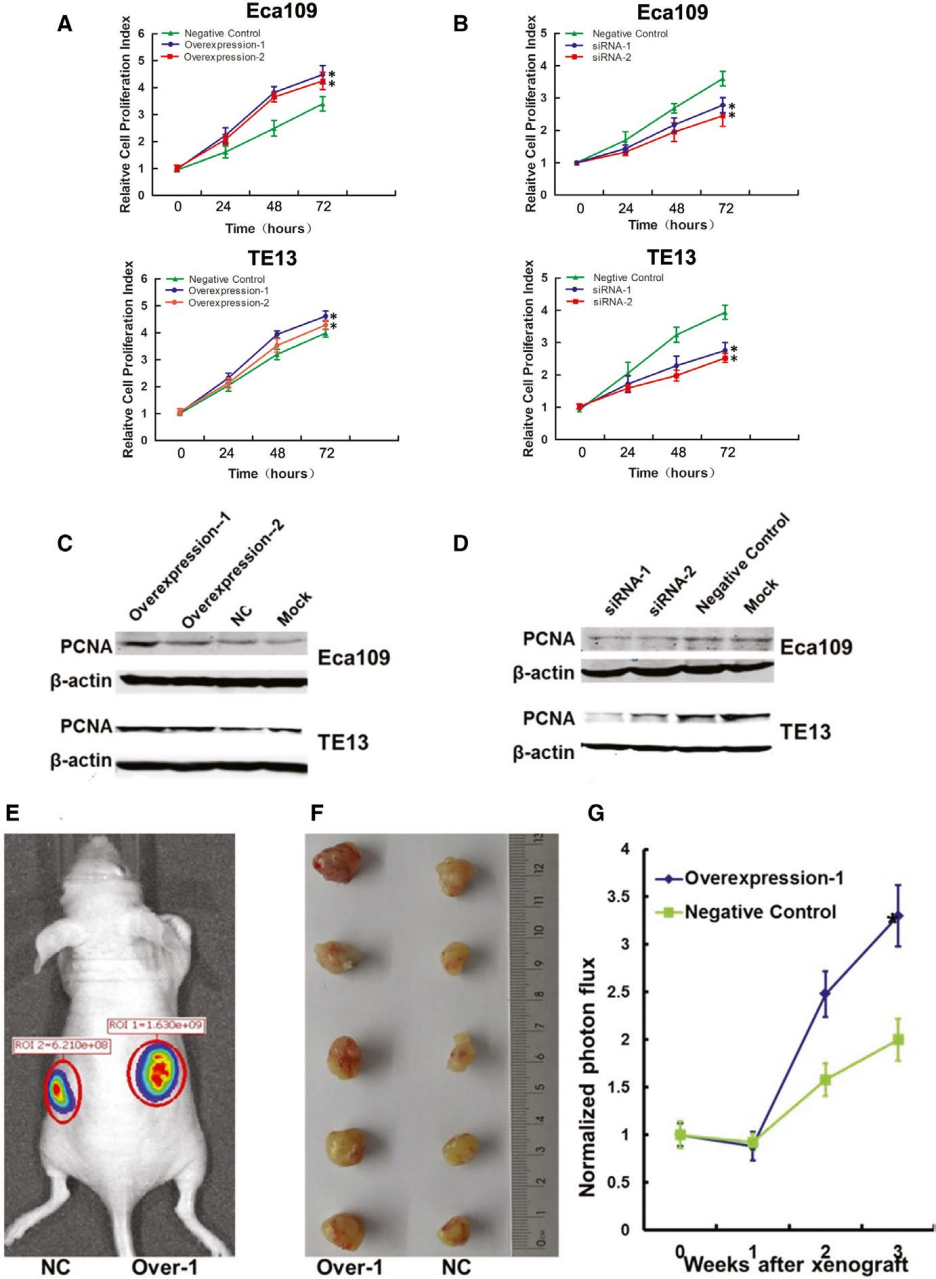
High Expression of lncRNA‐HEIH Promotes ESCC Cell Proliferation In Vitro and Vivo. Eca109 cells and TE13 cells stably transfected with lentivirus encoding lncRNA‐HEIH (A) and whose transfected with siRNA against lncRNA‐HEIH (B) were seeded in 96‐well plates, and cell proliferation was assessed daily for three days using the CCK‐8 assay. Changes in the proliferation marker PCNA were shown by western blotting analysis and normalized to β‐actin (C) and (D). The in vivo models used were xenograft transplanted nude mouse tumour models of human ESCC growth established with lncRNA‐HEIH up‐regulated Eca109 cells (Over‐1, overexpression‐1). (E) Photographs of tumours by imaging with the IVIS system. A representative luciferase signal was captured in each group at 3 weeks after the cell injection. A photograph of the tumours is also presented (F). (G) Effect of lncRNA‐HEIH on ESCC tumour growth. The asterisk indicates a significant change (*P* < .05). The data are the mean ± standard deviations

To evaluate the influence of lncRNA‐HEIH on the biological behaviours of oesophageal cancer cells, we constructed cell lines with lncRNA‐HEIH stable overexpression and down‐expression (Figure S1). Cell counting kit‐8 assays indicated that the proliferation of oesophageal cancer cells was increased in Eca‐109 cells and TE13 cells when lncRNA‐HEIH was overexpressed and reduced in both cells when lncRNA‐HEIH was knocked down (Figure 2A,B). Consistent with increased cell proliferation, exogenous expression of lncRNA‐HEIH promoted protein level of PCNA, a proliferating cell nuclear antigen in Eca‐109 and TE13 cells (Figure 2C). In contrast, Eca‐109 and TE13 cells in which lncRNA‐HEIH was knocked down had significantly lower protein levels of PCNA compared with control cells (Figure 2D). These results suggest that lncRNA‐HEIH is a key factor in regulating cell proliferation. To probe into the effects of lncRNA‐HEIH on cancer cell dynamics in vivo, lncRNA‐HEIH up‐regulated (Eca‐109 overexpression‐1) and control cells were injected into the bilateral armpit of nude mice. The results showed that the growth of tumours from lncRNA‐HEIH up‐regulated xenografts was significantly promoted tumour formation compared with that control cells (Figure 2E‐G).

### 3.3 A wide range of gene expressions was altered after lncRNA‐HEIH overexpression

**Figure 3:**
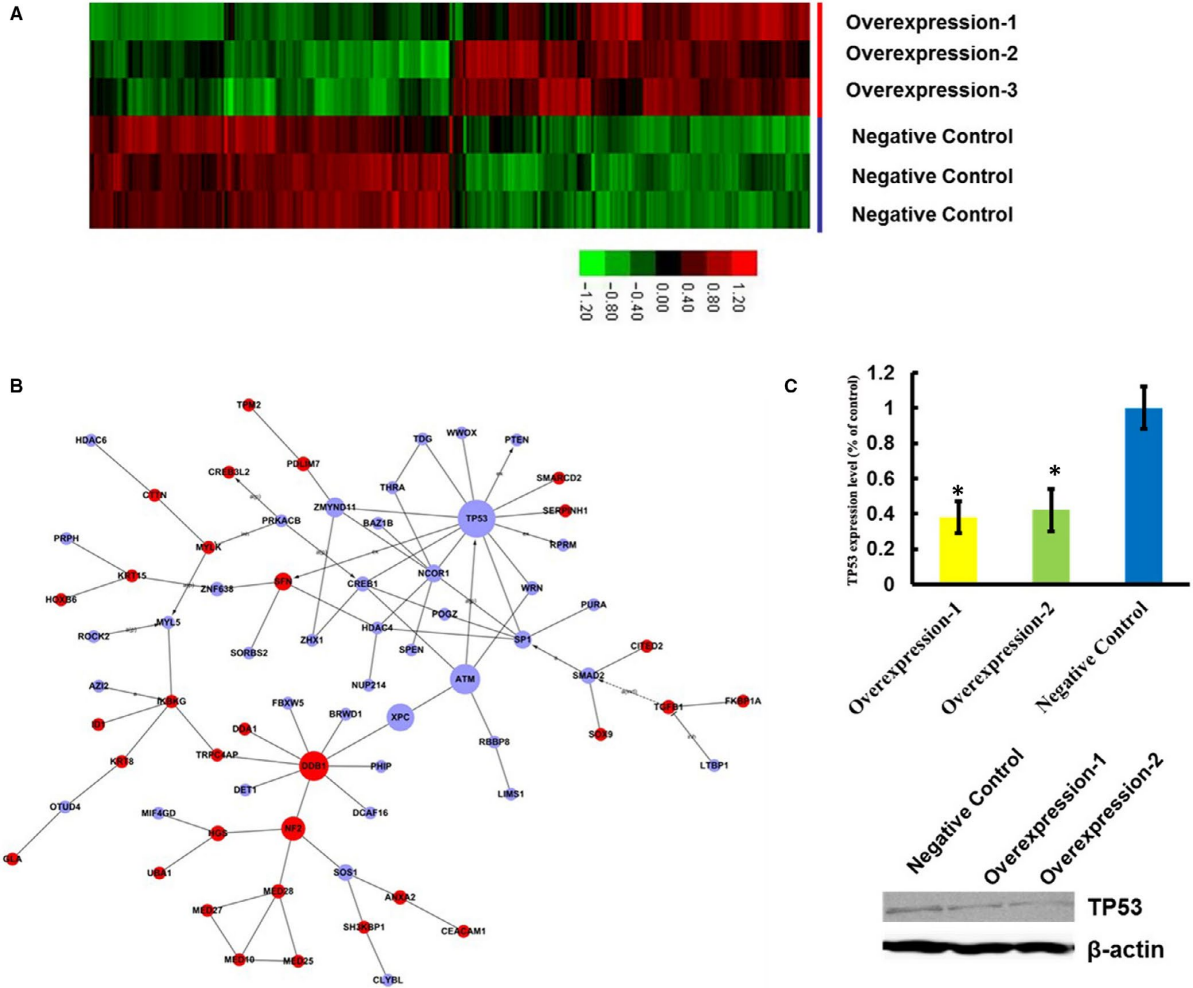
lncRNA‐HEIH Overexpression in ESCC Cell Induces Tp53 Down‐expression. (A) Heatmap of expression values from microarray shows differentially expressed genes in lncRNA‐HEIH overexpressed Eca109 cell. (B) Gene‐gene interaction network was constructed based on the data of differentially expressed genes. For instance, if there is confirmative evidence that two genes interact with each other, an interaction edge is assigned between the two genes. The considered evidence is the source of the interaction database from KEGG. Networks are stored and presented as graphs, where nodes are mainly genes and edges represent relation types between the nodes. Genes coloured in light blue are overexpressed genes in lncRNA‐HEIH overexpressed Eca109 cell. Genes coloured in red are down‐expressed genes. Node size represents the node degrees. Down‐expression of TP53 mRNA (C) and protein (D) level in lncRNA‐HEIH overexpressed Eca109 cell

DNA microarray data showed that lncRNA‐HEIH overexpression induced widespread changes in gene expression profile of cell line Eca‐109, with 449 genes up‐regulated and 419 down‐regulated (Figure 3A). The SAS system was used for gene ontology analysis (GO analysis) of the differentially expressed genes and *P* ≤ .05 was considered to be statistically significant (Table S3). Many of the identified genes are associated with signal transduction, metabolic process, cell apoptosis and cell cycle. Further analysis indicated that the lncRNA‐HEIH gene may participate in multiple signalling pathways (*P* < .01), such as p53 signalling pathway, MAPK signalling pathway and Wnt signalling pathway (Table S4).

Additionally, to determine the regulatory relationships of these differentially expressed genes and the key players in lncRNA‐HEIH‐related pathways, we performed a network analysis to generate an interaction network containing relevant biological information for the 449 up‐regulated and 419 down‐regulated genes. The resulting network shows a high degree of connectivity that further supported the existence of biologically related functions (Figure 3B). According to the connected subgraphs and their GO terms, the functional modules were enriched for the p53 signalling pathway. Some critical genes are located in these modules, including TP53 (Figure 3B), suggesting that TP53 may have important roles in the lncRNA‐HEIH regulated signalling pathways.

TP53 expression between lncRNA‐HEIH overexpression Eca‐109 and control cells was validated by RT‐PCR (Figure 3C). We found that there were significantly lower levels of TP53 in lncRNA‐HEIH overexpression Eca‐109 cells. We further performed Western blot analysis on TP53 (Figure 3D) and drew a conclusion that there was a good correlation between the protein level and the gene expression data.

### 3.4 LncRNA‐HEIH inhibiting TP53 expression by Polycomb Repressive Complex 2

**Figure 4:**
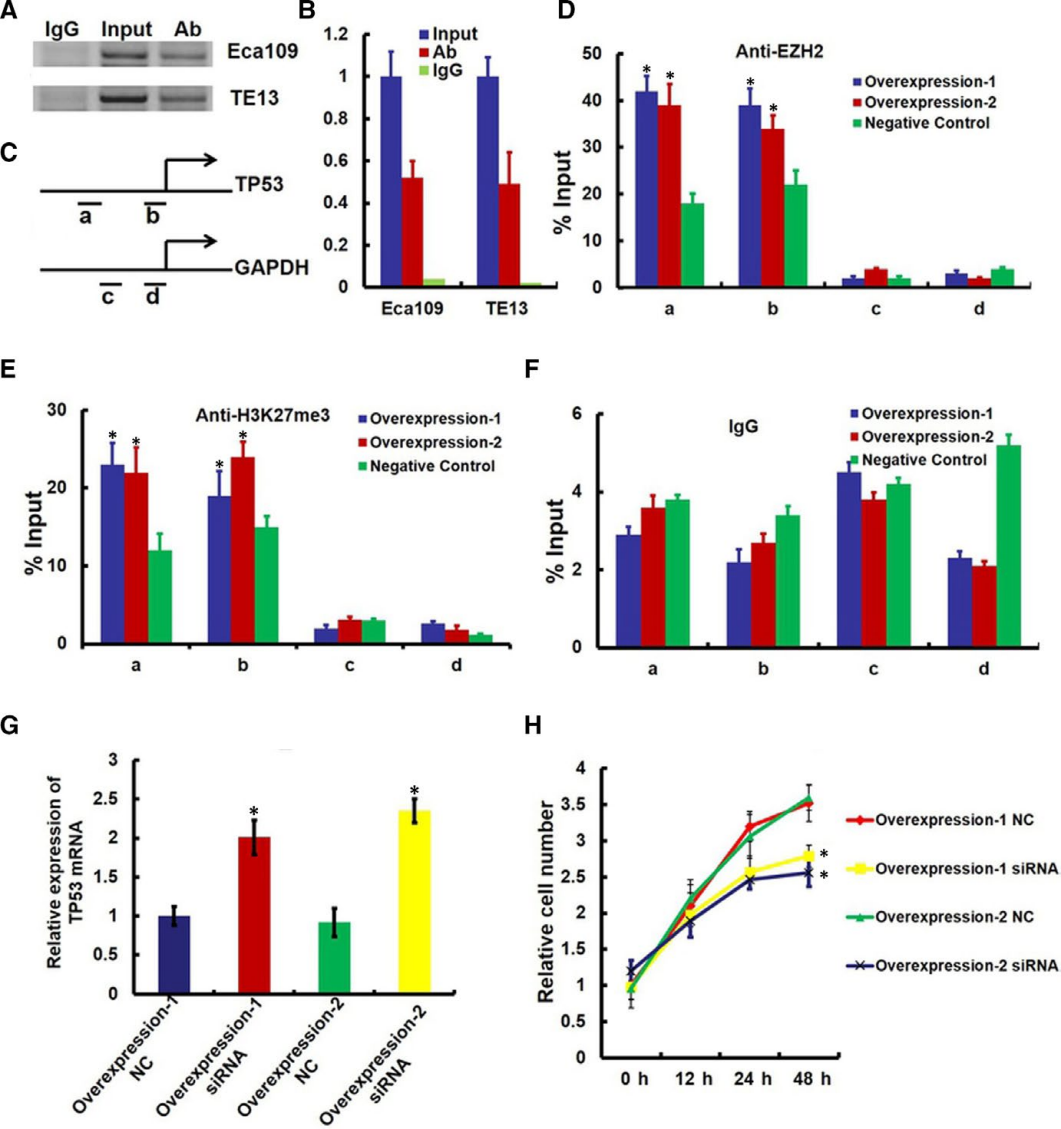
lncRNA‐HEIH‐induced down‐expression of TP53 requires EZH2. (A) RNA immunoprecipitation (RIP) experiments were performed using the EZH2 antibody to immunoprecipitate (IP) and a primer to detect lncRNA‐HEIH. (B) RIP enrichment was determined as lncRNA‐HEIH associated with EZH2 IP relative to an input control. (C‐F) ChIP analyses of lncRNA‐HEIH‐overexpressing Eca109 cells (Overexpression‐1 and Overexpression‐2) were conducted on TP53 (primer set a and b) and GAPDH (primer c and d) promoter regions using the indicated antibodies. Enrichment was determined relative to input controls. Asterisk indicates a significant change (*P* < .05). Data are the mean ± standard deviations of three independent biological replicates. (G) RT‐PCR analysis of TP53 in lncRNA‐HEIH‐overexpressing Eca109 cells simultaneously transfected with EZH2 siRNA. (H) Eca109 cells stably overexpression lncRNA‐HEIH and simultaneously transfected with EZH2 siRNA were seeded in 96‐well plates, and cell proliferation was assessed daily for 3 days using the Cell Counting Kit‐8 (CCK‐8) assay

Next, we investigated the mechanism by which lncRNA‐HEIH mediated reduced TP53 expression. lncRNA‐HEIH has been shown to physically associate with the polycomb repressive complex 2 (PRC2 complex), suggesting that it may have a general role in recruiting polycomb‐group proteins to TP53. For further investigation, we performed RNA immunoprecipitation (RIP) with an antibody against EZH2, which is an important subunit of PRC2 complex, from nuclear extracts of Eca109 and TE13 cells. We observed a significant enrichment of lncRNA‐HEIH with EZH2 antibody compared with the nonspecific IgG control antibody (Figure 4A and B). To address whether lncRNA‐HEIH is involved in transcriptional repression through recruitment of EZH2 to TP53 promoter, we conducted ChIP analysis in lncRNA‐HEIH overexpression Eca109 cells. ChIP arrays demonstrated lncRNA‐HEIH increased the binding of EZH2 and H3K27me3 levels across the TP53 promoter (Figure 4C‐F). Importantly, concomitant down‐regulation of EZH2 in lncRNA‐HEIH overexpressing cells reduced the proliferation rate to that of control cells (Figure 4G and H). These results demonstrate a functional relationship between lncRNA‐HEIH and PRC2 in inhibiting TP53 expression.

### 3.5 LncRNA‐HEIH inhibiting TP53 expression in ESCC tissues

**Figure 5:**
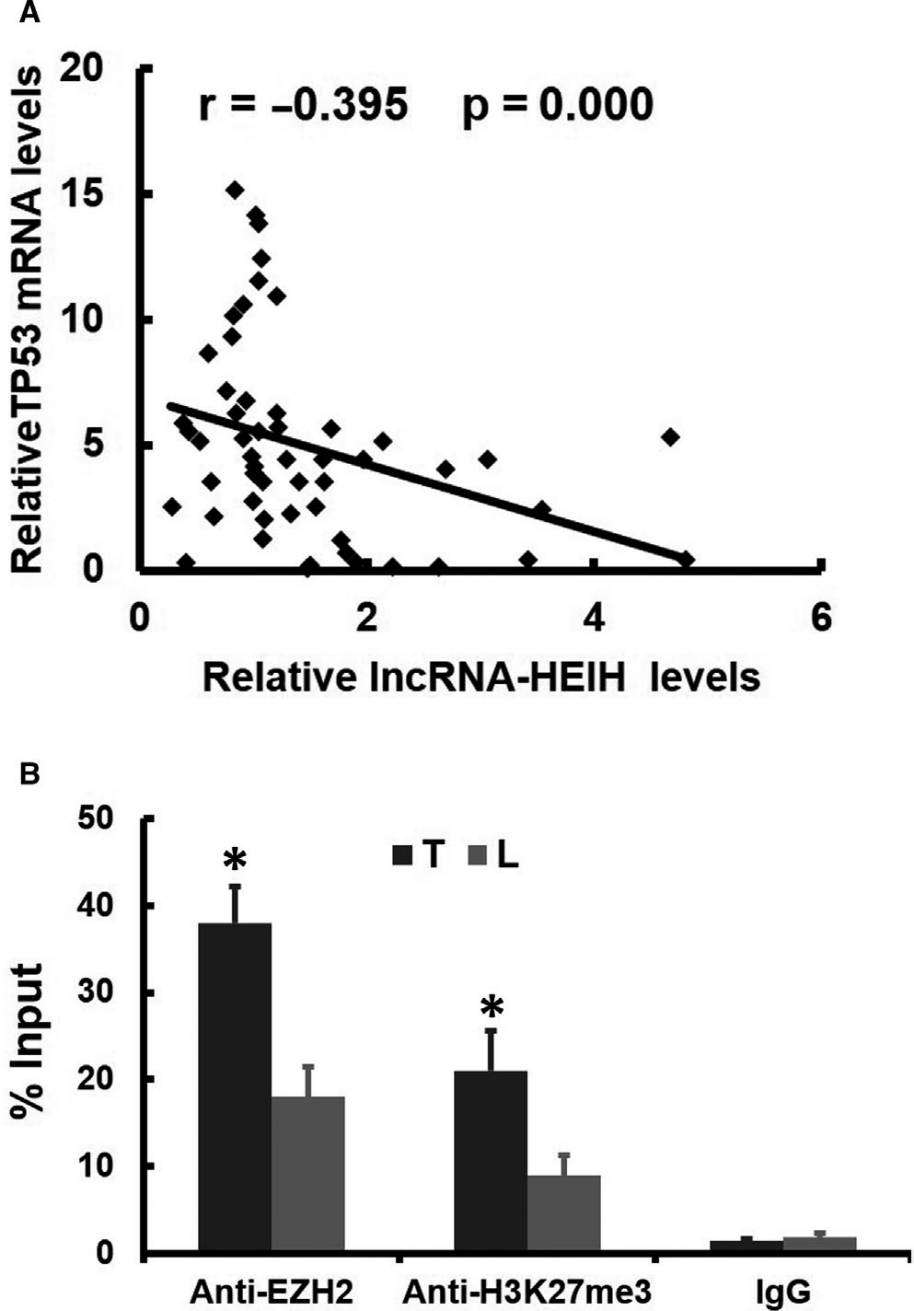
Validation in Clinical Samples. (A) Expression levels of lncRNA‐HEIH and TP53 were negatively correlated in ESCC samples, as measured by real‐time PCR. The relative expression values (normalized to β‐actin) were subjected to Pearson correlation analysis. (B) ChIP analyses of ESCC and adjacent tissues (n = 12) were conducted on the lncRNA‐HEIH promoter regions using anti‐EZH2 and H3K27me3. Enrichment was determined relative to input controls. The data are the means ± standard deviations of three independent experiments

We also investigated whether the mRNA expression of TP53 was inversely correlated with the levels of lncRNA‐HEIH in ESCC tissues. 91 primary ESCCs were analysed for their expression of both TP53 and lncRNA‐HEIH transcripts by RT‐PCR. A statistically significant inverse correlation was observed between TP53 and lncRNA‐HEIH transcripts (n = 41, *r* = −0.395, *P* = .000? by Pearson's correlation, Figure 5A). To determine whether TP53 is down‐regulated by increased histone methylation in vivo, we measured the enrichment of EZH2 and H3K27me3 levels across the TP53 promoter in 12 pairs of primary ESCC tissues (T) with matched adjacent tissues (L). In the 12 pairs of samples analysed, enrichment of EZH2 and H3K27me3 levels across the TP53 promoter was obviously increased in the ESCC samples compared to adjacent tissues (Figure 5B). However, we did not find any changes in enrichment of EZH2 and H3K27me3 levels across the GAPDH promoter region between the ESCC tissues and their pair‐matched adjacent tissues (Data not show).

## 4 DISCUSSION

LncRNAs have been estimated to regulate many human genes and control a variety of cellular processes.^14^ Recent studies have also shown that lncRNAs are deregulated in various cancers and their expression is relevant to the diagnosis and prognosis of a diverse array of tumours.^15^, ^16^ Even though various publications have studied the function of lncRNAs, the relation between lncRNA‐HEIH and ESCC remains unknown. In our study, we first demonstrated that lncRNA‐HEIH is significantly up‐regulated in human ESCC tissues. We also found that altered lncRNA‐HEIH expression levels are associated with the ESCC tumour invasion and clinical stage. We found that up‐regulation of lncRNA‐HEIH in Eca109 and TE19 cells could increase proliferative capability of these ESCC strains through suppressing the expression of TP53 gene. The observed aberrant expression of lncRNA‐HEIH suggests that it functions as an onco‐lncRNA in ESCC.

TP53 alterations, including mutation, have been identified as a frequent event in ESCC progression.^17^, ^18^ We analysed mutations in exons 5‐8 of the TP53 gene in selected cases, and ESCCs without missense mutation(s) or loss of heterozygosity (LOH) at the TP53 genomic locus were selected for expression association analysis of lncRNA‐HEIH and TP53. An interesting finding in this study was that aberrant expression of lncRNA‐HEIH was significantly correlated with expression of TP53 mRNA in ESCC tissue. In addition, we showed that the association of lncRNA‐HEIH with EZH2 could provide a hint to the complicated regulation mechanism of TP53 in ESCC. Overall, we proposed a model in which some lncRNAs associate with chromatin‐modifying complexes to regulate gene expression in ESCC.

Recently, many studies showed the function of lncRNAs as drivers of tumour oncogenic and play suppressive roles in ESCC.^19^, ^20^, ^21^ Previous reports have suggested that lncRNA‐HEIH was up‐regulated in hepatocellular carcinoma and associated with PRC2 to epigenetically regulate cell cycle‐related genes.^13^ In our study, we identified increased lncRNA‐HEIH levels in ESCC tissues versus non‐cancerous tissues by quick RT‐PCR. Additionally, the expression levels of lncRNA‐HEIH were up‐regulated in samples from patients with advanced clinical staging. These data indicated that up‐regulation of lncRNA‐HEIH was associated with the progression of ESCC. Moreover, we further studied the relationship between lncRNA‐HEIH expression and patient prognosis and discovered that increased lncRNA‐HEIH expression levels in ESCC corresponded remarkably to patient survival. The results manifested that high expression levels of lncRNA‐HEIH may play an important role in the tumorigenesis and progression of ESCC.

Taken together, the results of our study suggest that the expression of lncRNA‐HEIH correlates strongly with the clinical stages and overall survival times of ESCC patients, and that the up‐regulation of lncRNA‐HEIH plays a critical role in ESCC progression. Despite the need for further in‐depth exploration, including more animal experiments and subsequent clinical trials, our study still provides potential therapeutic strategies for ESCC treatment.

## CONFLICT OF INTEREST

The authors declare no conflict of interest.

## AUTHOR CONTRIBUTION

**XinYu Ding:** Conceptualization (equal); Data curation (equal); Formal analysis (equal); Investigation (equal); Methodology (equal); Resources (equal); Supervision (equal); Validation (equal); Visualization (equal); Writing‐original draft (equal); Writing‐review & editing (equal). **Chen Qi:** Data curation (equal); Formal analysis (equal); Funding acquisition (equal); Investigation (equal); Methodology (equal); Project administration (equal); Resources (equal); Software (equal); Supervision (equal); Validation (equal); Visualization (equal). **Jie Min:** Formal analysis (equal); Investigation (equal); Methodology (equal); Project administration (equal); Resources (equal); Software (equal); Supervision (equal); Validation (equal); Visualization (equal). **Zhifei Xu:** Investigation (equal); Methodology (equal); Project administration (equal); Resources (equal); Software (equal); Supervision (equal); Validation (equal); Visualization (equal). **KeNan Huang:** Investigation (equal); Project administration (equal); Resources (equal); Software (equal); Supervision (equal); Visualization (equal). **Hua Tang:** Funding acquisition (equal); Investigation (equal); Methodology (equal); Project administration (equal); Resources (equal); Validation (equal).

## Supporting information

Figure S1

Table S1

Table S2

Table S3

Table S4

## Data Availability

The data are free access to available upon request.
