# Supplementary figures and images for: Long non‐coding RNA HEIH suppresses the expression of TP53 through enhancer of zeste homolog 2 in oesophageal squamous cell carcinoma
Source: J Cell Mol Med. 2020 Jul 30;24(18):10551–9. doi: 10.1111/jcmm.15673 (PMC7521320; doi:10.1111/jcmm.15673)

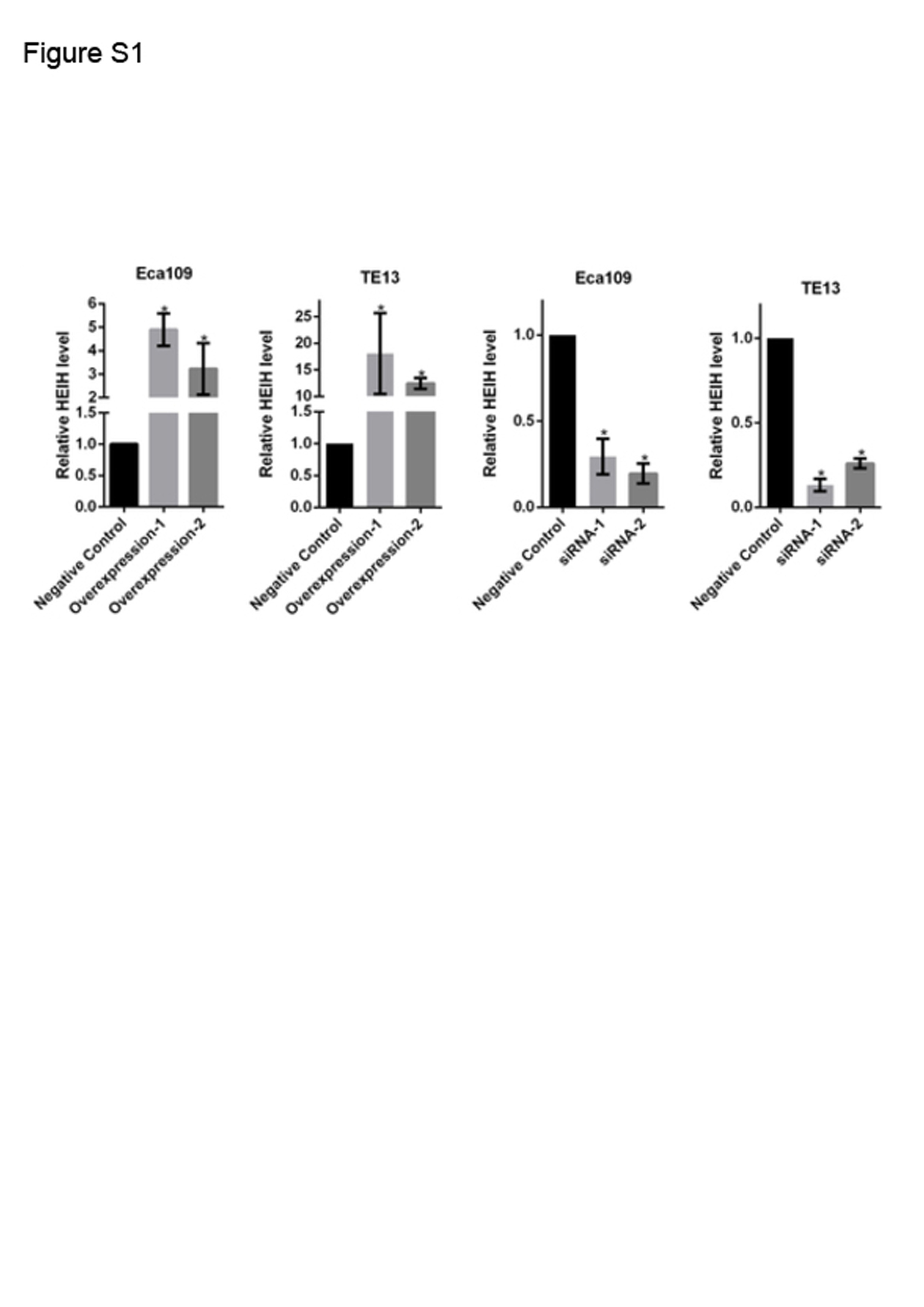

Supplement: Supplementary file 1 — Figure S1 [file JCMM-24-10551-s001.jpg]
