# Supplementary material for: Long non‐coding RNA HEIH suppresses the expression of TP53 through enhancer of zeste homolog 2 in oesophageal squamous cell carcinoma
Source: J Cell Mol Med. 2020 Jul 30;24(18):10551–9. doi: 10.1111/jcmm.15673 (PMC7521320; doi:10.1111/jcmm.15673)
Supplement: Supplementary file 2 — Table S1 [file JCMM-24-10551-s002.docx]

**Table S1.** lncRNA-HEIH siRNAs used in this study.

|  | Sequence |
| --- | --- |
| Negative control | 5' UUCUCCGAACGUGUCACGUTT 3' |
|  | 5' ACGUGACACGUUCGGAGAATT 3' |
| lncRNA-HEIH siRNA-1 | 5' GCCUUCCCUCUAACCUUAATT 3' |
|  | 5' UUAAGGUUAGAGGGAAGGCTT 3' |
| lncRNA-HEIH siRNA-2 | 5' GCCCAAAGCCACGUUUCUATT 3' |
|  | 5' UAGAAACGUGGCUUUGGGCTT 3' |
