# Supplementary material for: Long non‐coding RNA HEIH suppresses the expression of TP53 through enhancer of zeste homolog 2 in oesophageal squamous cell carcinoma
Source: J Cell Mol Med. 2020 Jul 30;24(18):10551–9. doi: 10.1111/jcmm.15673 (PMC7521320; doi:10.1111/jcmm.15673)
Supplement: Supplementary file 3 — Table S2 [file JCMM-24-10551-s003.docx]

**Table S2.** Primers used in this study.

|  | Sequence | Product size (bp) |
| --- | --- | --- |
| lncRNA-HEIH | 5' CCTCTTGTGCCCCTTTCTT 3' | 179 |
|  | 5' ATGGCTTCTCGCATCCTAT 3' |  |
| RIP primer | 5' GCAGCCTTGTGACAGTTCG 3' | 171 |
|  | 5' AGGTCAGGGTCACACTCTCG 3' |  |
| TP53 | 5' TGCTCAGATAGCGATGGTC 3' | 165 |
|  | 5' GTAGTTGTAGTGGATGGTGGTAC 3' |  |
| CHIP a | 5’TTCTCAATCCAGCAATCTATC3' | 208 |
|  | 5’CACCTACGACGAGGACACT3' |  |
| CHIP b | 5’CCAGCAGCCCTGAGGAGCAT3' | 165 |
|  | 5’AGGTCCTTCCCAGCATCCC3' |  |
| CHIP c | 5’GGTAGGGAGTTCGAGACCAG3' | 290 |
|  | 5’TCAACGCAGTTCAGTTAGGC3’ |  |
| CHIP d | 5’CCTTCTTGCCTTGCTCTTG3' | 197 |
|  | 5’GTTTAGCCTGCCTGGTGAT3' |  |
