# Supplementary material for: Long non‐coding RNA HEIH suppresses the expression of TP53 through enhancer of zeste homolog 2 in oesophageal squamous cell carcinoma
Source: J Cell Mol Med. 2020 Jul 30;24(18):10551–9. doi: 10.1111/jcmm.15673 (PMC7521320; doi:10.1111/jcmm.15673)
Supplement: Supplementary file 4 — Table S3 [file JCMM-24-10551-s004.docx]

| **Go ID** | **Name** | **Total** | **Hits** | **Percentage** | ***p* value** |
| --- | --- | --- | --- | --- | --- |
| GO:0007165 | Signal transduction | 1634 | 120 | 0.0734 | 0 |
| GO:0006139 | nucleic acid metabolic process | 1244 | 113 | 0.0908 | 0 |
| GO:0019538 | Protein metabolic process | 1231 | 115 | 0.0926 | 0 |
| GO:0006350 | transcription | 753 | 72 | 0.0956 | 0 |
| GO:0016070 | RNA metabolic process | 841 | 81 | 0.0963 | 0 |
| GO:0043412 | Biopolymer modification | 650 | 64 | 0.0985 | 0 |
| GO:0006629 | Lipid metabolic process | 325 | 46 | 0.1415 | 0 |
| GO:0006351 | Transcription dna dependent | 636 | 62 | 0.0975 | 1.11E-16 |
| GO:0051234 | Establishment of localization | 870 | 74 | 0.0851 | 2.22E-16 |
| GO:0048468 | Cell development | 577 | 58 | 0.1005 | 2.22E-16 |
| GO:0006950 | Response to stress | 508 | 54 | 0.1063 | 2.22E-16 |
| GO:0045449 | Regulation of transcription | 566 | 55 | 0.0972 | 6.55E-15 |
| GO:0007242 | Intracellular signaling cascade | 667 | 58 | 0.087 | 1.48E-13 |
| GO:0006915 | Apoptosis | 431 | 44 | 0.1021 | 6.21E-13 |
| GO:0007049 | Cell cycle | 315 | 37 | 0.1175 | 6.47E-13 |
| GO:0008283 | Cell proliferation | 513 | 44 | 0.0858 | 1.98E-10 |
| GO:0007243 | Protein kinase cascade | 293 | 29 | 0.099 | 1.05E-08 |

**Table S3**. Gene ontology analysis of differentially expressed genes
