# Supplementary material for: Long non‐coding RNA HEIH suppresses the expression of TP53 through enhancer of zeste homolog 2 in oesophageal squamous cell carcinoma
Source: J Cell Mol Med. 2020 Jul 30;24(18):10551–9. doi: 10.1111/jcmm.15673 (PMC7521320; doi:10.1111/jcmm.15673)
Supplement: Supplementary file 5 — Table S4 [file JCMM-24-10551-s005.docx]

| **Name** | **Total** | **Hits** | **Percentage** | **p-value** |
| --- | --- | --- | --- | --- |
| Pathways in cancer | 328 | 39 | 0.1189 | 1.04E-13 |
| MAPK signaling pathway | 267 | 31 | 0.1161 | 6.34E-11 |
| Focal adhesion | 201 | 26 | 0.1294 | 2.04E-10 |
| Lysosome | 121 | 19 | 0.157 | 2.08E-09 |
| Peroxisome | 78 | 14 | 0.1795 | 4.74E-08 |
| Endocytosis | 183 | 20 | 0.1093 | 4.01E-07 |
| ErbB signaling pathway | 87 | 13 | 0.1494 | 1.28E-06 |
| Gap junction | 90 | 13 | 0.1444 | 1.9E-06 |
| p53 signaling pathway | 69 | 11 | 0.1594 | 4.36E-06 |
| Regulation of actin cytoskeleton | 216 | 20 | 0.0926 | 5.35E-06 |
| Drug metabolism - cytochrome P450 | 72 | 11 | 0.1528 | 6.68E-06 |
| Progesterone-mediated oocyte maturation | 86 | 12 | 0.1395 | 6.77E-06 |
| TGF-beta signaling pathway | 86 | 12 | 0.1395 | 6.77E-06 |
| Proteasome | 48 | 9 | 0.1875 | 8.21E-06 |
| Apoptosis | 88 | 12 | 0.1364 | 8.63E-06 |
| Cell cycle | 128 | 14 | 0.1094 | 2.12E-05 |
| Wnt signaling pathway | 151 | 14 | 0.0927 | 0.00013 |
| Chemokine signaling pathway | 190 | 16 | 0.0842 | 0.000139 |
| Tight junction | 134 | 13 | 0.097 | 0.000142 |
| PPAR signaling pathway | 69 | 9 | 0.1304 | 0.000161 |

**Table S4.** Pathway analysis of differentially expressed genes
